# The role of the cancer testis antigen PRAME in tumorigenesis and immunotherapy in human cancer

**DOI:** 10.1111/cpr.12770

**Published:** 2020-02-05

**Authors:** Yichi Xu, Ruanmin Zou, Jing Wang, Zhi‐wei Wang, Xueqiong Zhu

**Affiliations:** ^1^ Departmant of Obstetrics and Gynecology The Second Affiliated Hospital of Wenzhou Medical University Wenzhou China; ^2^ Department of Pathology Beth Israel Deaconess Medical Center Harvard Medical School Boston MA USA

**Keywords:** immunotherapy, oncogene, PRAME, proliferation, tumorigenesis

## Abstract

Preferentially expressed antigen in melanoma (PRAME), which belongs to the cancer/testis antigen (CTA) gene family, plays a pivotal role in multiple cellular processes and immunotherapy response in human cancers. PRAME is highly expressed in different types of cancers and is involved in cell proliferation, apoptosis, differentiation and metastasis as well as the outcomes of patients with cancer. In this review article, we discuss the potential roles and physiological functions of PRAME in various types of cancers. Moreover, this review highlights immunotherapeutic strategies that target PRAME in human malignancies. Therefore, the modulation of PRAME might be useful for the treatment of patients with cancer.

## 1 INTRODUCTION

A cancer/testis antigen (CTA) termed preferentially expressed antigen in melanoma (PRAME) that encodes the human leucocyte antigen (HLA)‐A24 antigen was first characterized in 1997.^1^ The PRAME gene is localized on the reverse strand of chromosome 22 (22q11.22), is approximately 12 kilobases long and contains leucine‐rich repeat domains.^2^ PRAME, in addition to melanoma‐associated antigen (MAGE), B melanoma antigen (BAGE), G antigen (GAGE), New York oesophageal squamous cell carcinoma 1(NY‐ESO‐1) and L antigen family member 1 (LAGE‐1), belongs to the CTA gene family and encodes antigen peptides recognized by T lymphocytes.^3^ PRAME often has specific expression profiles: PRAME can be detected in many human malignancies; aside from its expression in the testes and limited expression in ovaries, adrenals and endometrium, PRAME is not detected in healthy human tissues.^1^ Male germline cells have genome‐wide demethylation, which leads to high expression of PRAME in the testes.^1^ It has been reported that the PRAME gene is hypermethylated in normal tissues; however, this gene is hypomethylated in most malignant cells. Ortmann et al observed that treatment with the demethylating agent 5′‐Aza‐2′dC contributes to a dose‐related increase in PRAME expression in PRAME‐negative U‐937 and THP‐1 cell lines with hypermethylation of PRAME, suggesting that treatment with the demethylating agents results in upregulation of PRAME expression in certain malignant cells.^4^, ^5^, ^6^

The PRAME gene encodes a membrane‐bound protein and causes autologous cytotoxic T cell‐mediated immune responses.^1^, ^7^ A group found that the overexpression of PRAME blocks retinoic acid (RA)‐mediated cell differentiation, cell growth arrest and apoptotic death, suggesting that PRAME appears to serve as an inhibitor of retinoic acid receptor (RAR) signalling.^8^ Hence, upregulation of PRAME contributes to tumorigenesis via inhibiting the RA/RAR signalling pathway.^8^, ^9^ In line with this, high expression of PRAME is observed in 88% of primary tissues and 95% of metastatic tissues in melanomas.^1^ In addition to melanoma, PRAME is frequently expressed in numerous solid cancers, such as head and neck cancer, breast cancer, renal cell carcinoma and non–small‐cell lung cancer (NSCLC).^10^, ^11^, ^12^, ^13^ Notably, it has been revealed that PRAME is absent in normal hematopoietic tissues.^7^, ^14^ Nevertheless, some findings have uncovered that a high expression level of PRAME exists in acute and chronic leukaemia as well as in Hodgkin's lymphomas.^7^, ^14^, ^15^ In addition, the expression level of PRAME is a prognostic biomarker for poor clinical outcomes in breast cancer and neuroblastoma.^16^, ^17^ However, the expression level of PRAME is associated with a better chemotherapy response and favourable survival in acute myeloid leukaemia (AML).^7^, ^18^ Therefore, the different roles of PRAME as an oncogene or a tumour suppressor gene in various malignancies may depend on the tumour types.

PRAME has been identified as a potential candidate for immunotherapy, eliciting a strong immune reaction in patients with AML, chronic myelogenous leukaemia (CML), acute lymphoblastic leukaemia (ALL) and melanoma.^19^, ^20^, ^21^, ^22^ Currently, the efficacy of anti‐PRAME vaccines is being evaluated for PRAME‐positive tumours in a clinical study.^23^ Therefore, in this review article, we describe the role of PRAME in tumorigenesis. Moreover, we highlight whether PRAME might serve as a useful prognostic biomarker in numerous human cancers. Furthermore, we discuss whether PRAME might be a promising target for potential immunotherapy in human malignant tumours.

## 2 ROLE OF PRAME IN CANCERS

### 2.1 Breast cancer

Globally, there were approximately 2.1 million newly diagnosed cases of female breast cancer in 2018, accounting for a quarter of cancer cases in females.^24^ The PRAME antigen is expressed widely among diverse breast cancer subtypes, including hormone‐sensitive tumours.^25^, ^26^ Moreover, PRAME expression is involved in poor clinical outcome and is useful in conjunction with clinical parameters to predict breast cancer outcomes.^16^, ^27^ Notably, the expression level of PRAME is correlated with negative oestrogen receptor status, lower rates of overall survival and elevated rates of distant metastases.^28^ It has been reported that PRAME could serve as a tumour‐promoting factor in triple‐negative breast cancer. Mechanistically, this phenomenon is due to the promotion of cancer cell motility through EMT‐related gene reprogramming.^29^ Moreover, Sun et al revealed that PRAME might be a biomarker candidate for breast cancer.^30^ Controversially, another report revealed that PRAME inhibits the proliferation and metastasis of breast cancer cells, demonstrating that PRAME might be a tumour suppressor in breast cancer.^31^ Thus, due to the limited research on the functions of PRAME in breast cancer, it is necessary to further determine the role of PRAME in the development and progression of mammary malignancy.

### 2.2 Cervical cancer

Cervical cancer is responsible for 570 000 cancer cases worldwide, ranking fourth in incidence in 2018.^24^ A study showed that overexpression of PRAME in HeLa cervical cancer cells leads to an apparent change in morphology.^32^ Concomitantly, PRAME‐transfected HeLa cells show cytoplasmic vacuolization and blebbing. Later, HeLa cells disintegrated into apoptotic bodies, suggesting that PRAME might act as a tumour suppressor gene in cervical cancer progression.^32^

### 2.3 Haematological malignancies

Haematological malignancies comprise three major groups: leukaemia (acute leukaemia and CML), B and T/natural killer (NK) cell lymphomas, and plasma cell malignancy (multiple myeloma, MM).^33^ Numerous investigations have shown that increased PRAME expression is associated with poor outcomes, drug resistance and disease progression in chronic leukaemia (CL), MM, Hodgkin's lymphoma (HL) and diffuse large B‐cell lymphoma (DLBCL) patients.^34^, ^35^, ^36^, ^37^ For instance, in DLBCL patients treated with rituximab, cyclophosphamide, hydroxydaunorubicin, oncovin and prednisone (R‐CHOP) therapy, higher expression of PRAME is associated with shorter overall survival (OS) and shorter progression‐free survival (PFS) indicating that PRAME expression level might be a novel prognostic factor for DLBCL patients treated with R‐CHOP therapy.^36^ Additionally, PRAME is highly expressed in Down's syndrome‐acute megakaryoblastic leukaemia (DS‐AMKL) patients who are more likely to progress, but not in Down's syndrome‐transient myeloproliferative disorder (DS‐TMD) patients that are likely to self‐regress. This suggests that PRAME might be a potentially ideal indicator for the distinction between DS‐AMKL and DS‐TMD cases.^38^ Furthermore, it is well known that Wilms’ tumour gene 1 (WT1) gene is a tumour biomarker for a wide variety of haematological malignancies.^39^ Hence, the combination of these two tumour biomarkers might cover a broad range of patients with leukaemia.^40^ Furthermore, malignant plasma cells from the majority of MM patients express MAGE‐1, MAGE‐3 and PRAME. However, polyclonal reactive plasma cells do not express any of these genes.^41^ Another study found that expression of ETS‐related genes (ERGs), ecotropic viral integration site‐1 (EVI1) and PRAME could allow a greater distinction between AML and cytogenetically normal AML (CN‐AML) patients, which could be useful for improving their individual prognostic significance and patient risk stratification.^42^ In the CML cell line model, PRAME overexpression inhibits RAR‐mediated cell differentiation, growth arrest and apoptosis. When the PRAME/RAR effect is blocked, CML cells can differentiate and undergo apoptotic death, even when CML is in the advanced phase.^8^ Consistent with this finding, Tanaka et al found that PRAME expression is associated with cell cycle progression from the G0/G1 phase to the S phase.^43^ Overexpression of PRAME also leads to the suppression of apoptosis and the blockade of erythroid differentiation in the CML cell line.^43^ In support of the oncogenic role of PRAME, one report demonstrated that knockdown of PRAME could evoke the expression of tumour necrosis factor‐related apoptosis‐inducing ligand (TRAIL) and increase the sensitivity of CML cells to imatinib, suggesting that PRAME may be associated with tumour progression via blockade of the TRAIL pathway.^44^

PRAME hypomethylation might lead to its increased expression in CML blast crisis and AML, resulting in an enhancement of the oncogenic activity of PRAME.^4^, ^5^ Controversially, there are several reports showing that PRAME may be a tumour suppressor in haematological malignancies. One study uncovered that PRAME expression is associated with a better outcome in haematological malignancies such as AML and ALL.^42^, ^45^, ^46^ It has been found that a high level of PRAME expression has a favourable prognosis in leukaemia, and PRAME overexpression increases leukaemia cell apoptosis and suppresses proliferation by downregulation of S100A4 and upregulation of p53.^47^, ^48^ Tajeddine et al indicated that overexpression of PRAME causes a decrease in the expression of heat shock protein 27 (Hsp27) and S100A4 at the transcriptional level, thus correlating with a better prognosis in leukaemia. In animal models, downregulation of PRAME induces tumour growth in leukaemia.^32^ Altogether, the high expression level of PRAME could have a favourable prognosis in acute leukaemia.

Haematological malignancies such as CML and AML as well as lymphomas could be vulnerable to control by the immune system, and effector T cells, which recognize minor histocompatibility antigens and tumour‐associated antigens (TAAs) that are overexpressed in tumour cells, play a key role in this process.^49^, ^50^, ^51^, ^52^, ^53^ PRAME is overexpressed in many haematological malignancies but is absent in normal tissues. Therefore, it may be a suitable candidate for T cell‐mediated immunotherapy.^54^ Strikingly, PRAME‐specific cytotoxic T lymphocyte (CTL) lines induce high‐avidity CTLs that do not affect normal hematopoietic progenitors, indicating that this could be valuable for immunotherapy of haematological malignancies with high expression of PRAME.^54^ PRAME could be a potential target antigen for adoptive T‐cell therapy and vaccination of patients with CML.^19^ HLA‐B62‐restricted PRAME peptides are presented on adult T‐cell leukaemia (ATL) cells, and CTLs induced by this peptide that were obtained from healthy volunteers were able to kill PRAME‐positive ATL cells.^55^ Moreover, the antigen receptors for hyaluronic acid‐mediated motility (RHAMM), PRAME, M‐phase phosphoprotein 11 (MPP11) and G250 might be candidates for immunotherapies of leukaemia patients. Because of their simultaneous expression, these antigens also constitute targets for polyvalent vaccines.^22^, ^23^ Therefore, CTLs in combination with peptide vaccines could maintain long‐term immune surveillance. Similar to these reports, PRAME and other HL‐associated CTAs might be useful for monitoring HL‐directed immune responses or as targets for HL‐specific immunotherapy.^56^ Additionally, PRAME is highly expressed in chemoresistant HL cells, indicating that immunotherapy might be a promising approach for patients with chemoresistant HL.^57^ PRAME‐positive AML dendritic cells (DCs) are recognized by specific T cells, which might be a powerful tool for AML immunotherapy.^58^ Several studies have shown that PRAME could be an attractive target for monitoring minimal residual disease (MRD).^59^, ^60^, ^61^ On the other hand, patients with positive PRAME expression and MRD after hematopoietic stem cell transplantation (HSCT) treatment do not respond well to pre‐emptive immunotherapy. Choosing appropriate interventions can further improve the clinical outcomes of PRAME‐ and MRD‐positive patients.^62^ One study found that epigenetic upregulation of PRAME by a demethylating agent could lead to increased expression of PRAME, suggesting that epigenetic regulators in combination with specific CTA can improve the effect of immunotherapy in patients with AML.^63^ These results demonstrate that the PRAME protein could be processed by a demethylating agent and present on the cell surface of leukaemia cells and might be the target antigen for immunotherapy in leukaemia patients.

### 2.4 Lung cancer

Lung cancer remains the primary cause of cancer morbidity and mortality worldwide, with 2.1 million new cases and 1.8 million deaths in 2018.^24^ PRAME has been identified as an essential player in the development of NSCLC.^64^ In NSCLC patients, it has been reported that higher levels of MAGE‐A3 and PRAME expression are found in squamous cell carcinomas compared to adenocarcinomas, as well as in smokers compared to nonsmokers.^13^ Similarly, another study reported that the expression of PRAME and MAGE‐A3 is more frequent in NSCLC squamous cell carcinomas than in adenocarcinomas.^65^ These results indicate that high expression of PRAME might be considered during the clinical development of antigen‐specific cancer immunotherapy. PRAME expression is decreased in lung adenocarcinoma and lung bone metastasis tissues compared to normal lung tissues.^66^ In addition, in vitro experiments revealed that downregulation of PRAME could promote the metastasis of lung cancer cells, suggesting that PRAME might play a key role in preventing the progression and metastasis of lung adenocarcinoma.^66^

Non–small‐cell lung cancer patients treated with recombinant PRAME protein exhibit a humoral response and thereby stimulate CD4‐positive responses and enhance anti‐tumour activity.^67^ The percentage of patients with PRAME‐specific CD4‐positive T cells at a dose of 500 µg of PRAME is higher than that at lower doses.^67^ At the same time, it has been shown that the PRAME immunotherapeutic dose of 500 µg is a safe and clinically acceptable dose.^67^ Similarly, PRAME‐derived peptides trigger frequent specific T‐cell responses in patients with lung cancer and are an appropriate candidate for targeted immunotherapy.^68^ Moreover, in the adjuvant setting, the most relevant and promising vaccine directly targeting PRAME could be a potential therapeutic approach for NSCLC patients.^69^

### 2.5 Melanoma

Melanoma is a common deadly cancer, with approximately 90 000 new invasive cases and approximately 10 000 deaths per year.^70^ Increasing evidence supports the important roles of PRAME in melanoma tumorigenesis. It has been reported that PRAME is highly overexpressed in melanoma tumour samples and that PRAME is considered an immunotherapy target for the treatment of melanoma.^71^, ^72^ PRAME‐positive status is significantly correlated with the largest basal diameter (LBD), tumour volume and worsening gene expression profiling (GEP) class in uveal melanoma (UM).^73^ Two studies confirmed that PRAME is significantly associated with an increased risk of metastasis in uveal melanomas.^74^, ^75^ In addition, one study confirmed that the prognostic accuracies of GEP and PRAME are superior to that of tumour‐node‐metastasis (TNM) staging in UM. Furthermore, GEP combined with PRAME enhanced the prognostic accuracy of the molecular prognostic model.^76^

One study revealed that downregulation of miR‐211 triggered elevated PRAME expression in melanoma cells.^77^ In addition, DNA methylation is negatively correlated with PRAME expression in melanoma cells. After treatment with a DNA methylation inhibitor, the expression of myeloid zinc finger 1 (MZF1) and PRAME significantly increases at both the protein and mRNA levels. Moreover, MZF1 promotes PRAME expression, leading to enhancement of the colony‐forming capability of melanoma cells.^78^ Because of the heterogeneous expression of CTAs including PRAME in human cutaneous melanoma, the DNA hypomethylating agent 5‐AZA‐dCyd could reverse the CTA‐negative and weakly positive phenotype of different melanoma cells in tumour lesions, producing a group of tumour cells homogeneously expressing therapeutic CTA to be targeted by CTA‐specific CTL.^79^ Gezgin et al noticed that PRAME‐specific T cells efficiently recognized UM cell lines expressing PRAME, suggesting that PRAME‐directed immunotherapy might play a potential role in selected patients with metastatic UM.^72^ Notably, in the phase I clinical study of melanoma patients, the PRAME immunotherapeutic approach using intramuscular injections of the recombinant PRAME protein with AS15 immunostimulant exhibits an acceptable safety profile and triggers similar anti‐PRAME–specific cellular and humoral immune responses.^80^

### 2.6 Ovarian cancer

In 2018, ovarian cancer was one of the most deadly malignancies in women, with 295 414 new ovarian cancer cases and 184 799 deaths.^24^ PRAME expression is increased in ovarian cancer tissues compared with normal ovarian tissues.^81^, ^82^ For instance, PRAME is frequently expressed in epithelial ovarian cancer at the mRNA and protein levels due to demethylation of PRAME, and its DNA methylation level is inversely correlated with its expression.^83^ Several possible tumour‐associated antigens, mucin 1 (MUC1), MUC20, folate receptor 1 (FOLR1) and PRAME have elevated expression levels in all high‐grade serous ovarian cancer cell lines.^84^ Furthermore, PRAME expression is absent in mesothelial cells lining the peritoneal cavity and in the fibroblasts of ovarian cancer patients.^84^ The expression level of PRAME is high in tumours from deceased patients, indicating that PRAME might act as a poor prognostic factor in patients with late‐stage serous ovarian adenocarcinoma.^85^ Consistent with this study, Partheen et al showed a similar result, in which PRAME expression is lower in ovarian cancer tissues from survivors compared with tissues from deceased patients.^86^ However, one group found that the expression of PRAME was not significantly different between survivors and deceased ovarian cancer patients.^87^ Thus, the different results of the two studies indicate that the function of PRAME needs to be further explored in ovarian cancer.

### 2.7 Sarcoma

Sarcomas consist of more than one hundred diverse bone and soft tissue cancers that are rare and heterogeneous, accounting for approximately 1% of adult tumours and 15% of paediatric cancers.^88^ The antigen PRAME is highly expressed in numerous types of sarcomas, such as synovial sarcoma, liposarcomas and myxoid/round cell liposarcoma.^89^, ^90^, ^91^, ^92^ Moreover, myxoid liposarcomas have higher levels of PRAME and NY‐ESO‐1 than other liposarcomas at both the transcriptional and translational levels. Their expression levels are positively associated with tumour grade and poor prognosis.^91^ By microarray dataset analysis, hypomethylation of PRAME is observed in osteosarcoma (OS), which leads to an increase in PRAME expression contributing to OS progression, indicating that PRAME could be useful for cancer diagnosis and treatment.^93^ Additionally, PRAME is homogeneously expressed in OS tissue, and knockdown of PRAME inhibits cell proliferation and colony formation and causes cell cycle arrest at G1 phase.^94^ In addition, one study indicated that PRAME is one of the candidate antigens in Ewing sarcoma (EwS), but even under optimal conditions, EwS‐associated antigens do not induce effective T‐cell receptor (TCR)‐mediated anti‐tumour immune responses. Hence, TCR engineering strategies could provide a more effective means to manipulating T‐cell immunity to target tumour cell elimination.^95^ The expression of NY‐ESO‐1 and PRAME could be induced by 5‐Aza‐dC treatment in chondrosarcoma cell lines, including in cell lines with absent or almost undetectable expression.^96^ These findings indicate that with adoptive immunotherapy following 5‐Aza‐dC treatment, NY‐ESO‐1/LAGE‐1s and PRAME‐specific CD8+ effector T cells can treat chondrosarcoma, which might be a promising way to treat patients with unresectable or metastatic chondrosarcoma.^96^

### 2.8 Other tumours

An elevated expression level of PRAME is found in hepatocellular carcinoma (HCC), and patients with a higher level of PRAME expression have a poorer prognosis in HCC, indicating the potential for the use of PRAME as a biomarker for unfavourable prognosis in HCC.^97^ Furthermore, PRAME expression is positively correlated with alpha fetoprotein levels, tumour size and American Joint Committee on Cancer (AJCC) clinical tumour stage in HCC.^97^ Biologically, the decreased expression of PRAME inhibits cell growth and induces cell apoptosis by activating p53/B‐cell lymphoma 2 (Bcl‐2)‐mediated apoptosis pathway and increasing p21 expression.^97^ PRAME is also expressed at a high frequency in head and neck squamous cell carcinoma (HNSCC) cell lines and HNSCC tissues.^98^ In addition, PRAME expression is positively associated with clinicopathologic markers of poor outcome in HNSCC,^98^ and PRAME expression is always present in metastatic lymph nodes of HNSCC.^10^ In addition, elevated PRAME expression is found in medulloblastoma (MB), and its higher expression indicates poorer prognosis in MB.^99^ Further mouse modelling studies found that tumour growth could efficiently be controlled in MB by using genetically modified T cells with a PRAME‐specific TCR. PRAME‐specific TCRs might represent an attractive novel approach for treating patients with MB.^99^ A high frequency of PRAME expression is observed in salivary duct carcinoma (SDC) but not in normal salivary gland tissues, immune cells or stromal cells.^100^ The high frequency and selective expression of PRAME in tumour tissues make PRAME a promising diagnostic biomarker for monitoring malignancy and might be an attractive target for cancer vaccination in SDC.^100^ In prostate cancer, it has been uncovered that PRAME is one of the downstream targets of miR‐421, and miR‐421 binds to the 3′‐untranslated region (UTR) of PRAME to inhibit the expression of PRAME.^101^ One group revealed that PRAME acts downstream of SRY‐box transcription factor 17 (SOX17) by repressing the regulation of germ cell differentiation and pluripotency in seminomas.^102^ Additionally, it has been reported that PRAME expression is associated with a poor response to chemotherapy in bladder urothelial carcinoma patients.^103^ These studies reveal that PRAME might be a carcinogenic gene and that targeting the PRAME oncoprotein might be a promising anti‐cancer therapeutic strategy.

## 3 CONCLUSION AND PERSPECTIVE

**Figure 1:**
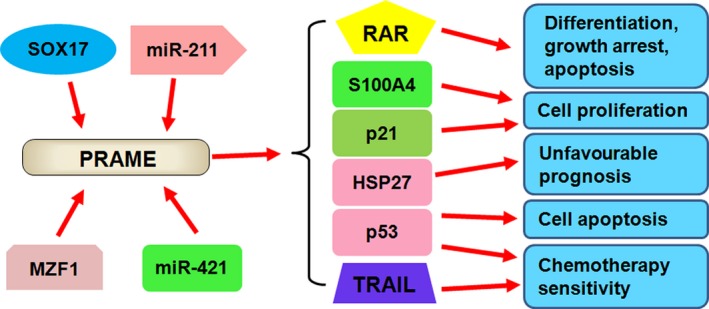
PRAME is regulated by upstream molecules and exerts its biological functions via the regulation of downstream targets in cancer. SOX17, MZF1, miR‐211 and miR‐421 regulate the expression of PRAME. PRAME exerts its biological functions via targeting p21, p53, RAR, TRAIL, S100A4 and HSP27, leading to the control of several cellular processes, including cell proliferation, apoptosis, differentiation, growth arrest and chemotherapy sensitivity

In summary, PRAME is not only expressed in the normal testis but is also widely expressed in numerous cancers. Moreover, PRAME can act as an oncogene or a tumour suppressor gene in different cancer types (Table 1). PRAME exerts its biological functions via regulation of its downstream targets, such as p53, p21, Bcl‐2, TRAIL, RAR, Hsp27 and S100A4 in human malignancies (Figure 1). Notably, PRAME is critical in the immunotherapy response and may be an attractive target for human cancer immunotherapy. Indeed, several clinical trials of PRAME immunotherapies have shown their safety and potent immune responses in melanoma, lung cancer and other advanced solid tumours.^67^, ^80^, ^104^ Antigen delivery and target specificity might affect the efficacy of PRAME immunotherapy. In addition, we have described a number of modulators, such as demethylating agents, to improve the effect of PRAME‐based immunotherapy in human malignancies.

**Table 1:** Role of PRAME in human cancers

Cancer type	Function	Target	References
Breast cancer	Involved in poor survival and distant metastases; relates with negative oestrogen receptor status	N/A	16, 27, 28
Cervical cancer	Associates with cell apoptosis	N/A	32
Haematological malignancies	Inhibits cell differentiation, growth arrest and apoptosis; increases the sensitivity to chemotherapy; and promotes cell apoptosis and favourable prognosis	Inhibits RAR signal and TRAIL; promotes p53; and downregulates S100A4, HSP27 and p21	8, 32, 44, 45
Lung cancer	Exhibits higher expression in squamous cell carcinomas lung cancer patients than adenocarcinomas	N/A	65
Melanoma	Associates with an increased risk of metastasis; enhances cell colony‐forming capability	Promotes by MZF1 and inhibits by miR‐211	74, 75, 77, 78
Ovarian cancer	Exhibits higher expression in tumours from deceased patients, and its function remains unknown	N/A	85, 86
Sarcoma	Associates with tumour grade and poor prognosis; inhibits cell proliferation and colony formation; and causes cell arrest at G1 phase	N/A	91, 94
Other tumours			
Seminomas	Regulation of cell differentiation and pluripotency	Activates by SOX17	102
HCC	Correlates with alpha fetoprotein levels, tumour size, AJCC stage and poor survival prognosis; induces cell growth; and inhibits cell apoptosis	Inhibits p53/Bcl2 and p21	97
HNSCC, MB, prostate cancer and bladder carcinoma	Related to poor prognosis, but function remains unknown; associates with poor response to chemotherapy	Inhibits by miR‐421	98, 99, 101, 103

Although the function of PRAME has been discussed, there are still several remaining questions. For instance, what are the upstream signalling pathways that regulate PRAME? How does PRAME govern their downstream targets? What other innovative technologies can improve the effect of PRAME‐based immunotherapy? To address these issues, we will need to use whole‐body or tissue‐specific knockout or knock‐in mouse models that will help us determine the carcinogenic effects of PRAME in human cancers. Altogether, in‐depth studies could contribute to the development of novel countermeasures and innovative technologies to target PRAME in immunotherapy for fighting human malignancies.

## ACKNOWLEDGEMENTS

This work was supported by the Science and Technology Planning Project of Wenzhou City (No. ZS2017006).

## CONFLICT OF INTEREST

The authors declare that they have no conflict of interest.

## AUTHOR CONTRIBUTIONS

Y.X. searched the literature and wrote the manuscript. R.Z. and J.W. made the figure and table. ZW.W. and X.Z. critically viewed, edited and approved the manuscript.

## Data Availability

The data that support the findings of this study are available from the corresponding author upon reasonable request.

## References

[1] Ikeda H , Lethé B , Lehmann F , et al. Characterization of an antigen that is recognized on a melanoma showing partial HLA loss by CTL expressing an NK inhibitory receptor. Immunity. 1997;6(2):199‐208. 9047241 10.1016/s1074-7613(00)80426-4

[2] Wadelin F , Fulton J , McEwan PA , Spriggs KA , Emsley J , Heery DM . Leucine‐rich repeat protein PRAME: expression, potential functions and clinical implications for leukaemia. Mol Cancer. 2010;9:226. 20799951 10.1186/1476-4598-9-226 PMC2936344

[3] Goodison S , Urquidi V . The cancer testis antigen PRAME as a biomarker for solid tumor cancer management. Biomark Med. 2012;6(5):629‐632. 23075240 10.2217/bmm.12.65

[4] Ortmann CA , Eisele L , Nückel H , et al. Aberrant hypomethylation of the cancer‐testis antigen PRAME correlates with PRAME expression in acute myeloid leukemia. Ann Hematol. 2008;87(10):809‐818. 18587578 10.1007/s00277-008-0514-8

[5] Roman‐Gomez J , Jimenez‐Velasco A , Agirre X , et al. Epigenetic regulation of PRAME gene in chronic myeloid leukemia. Leuk Res. 2007;31(11):1521‐1528. 17382387 10.1016/j.leukres.2007.02.016

[6] Schenk T , Stengel S , Goellner S , Steinbach D , Saluz HP . Hypomethylation of PRAME is responsible for its aberrant overexpression in human malignancies. Genes Chromosomes Cancer. 2007;46(9):796‐804. 17534929 10.1002/gcc.20465

[7] van Baren N , Chambost H , Ferrant A , et al. PRAME, a gene encoding an antigen recognized on a human melanoma by cytolytic T cells, is expressed in acute leukaemia cells. Br J Haematol. 1998;102(5):1376‐1379. 9753074 10.1046/j.1365-2141.1998.00982.x

[8] Epping MT , Wang L , Edel MJ , Carlee L , Hernandez M , Bernards R . The human tumor antigen PRAME is a dominant repressor of retinoic acid receptor signaling. Cell. 2005;122(6):835‐847. 16179254 10.1016/j.cell.2005.07.003

[9] Epping MT , Wang L , Plumb JA , et al. A functional genetic screen identifies retinoic acid signaling as a target of histone deacetylase inhibitors. Proc Natl Acad Sci USA. 2007;104(45):17777‐17782. 17968018 10.1073/pnas.0702518104 PMC2077016

[10] Figueiredo DL , Mamede RC , Proto‐Siqueira R , Neder L , Silva WA Jr , Zago MA . Expression of cancer testis antigens in head and neck squamous cell carcinomas. Head Neck. 2006;28(7):614‐619. 16475205 10.1002/hed.20380

[11] van't Veer LJ , Dai H , van de Vijver MJ , et al. Gene expression profiling predicts clinical outcome of breast cancer. Nature. 2002;415(6871):530‐536. 11823860 10.1038/415530a

[12] Neumann E , Engelsberg A , Decker J , et al. Heterogeneous expression of the tumor‐associated antigens RAGE‐1, PRAME, and glycoprotein 75 in human renal cell carcinoma: candidates for T‐cell‐based immunotherapies? Cancer Res. 1998;58(18):4090‐4095. 9751617

[13] Thongprasert S , Yang PC , Lee JS , et al. The prevalence of expression of MAGE‐A3 and PRAME tumor antigens in East and South East Asian non‐small cell lung cancer patients. Lung Cancer. 2016;101:137‐144. 27794402 10.1016/j.lungcan.2016.09.006

[14] Radich JP , Dai H , Mao M , et al. Gene expression changes associated with progression and response in chronic myeloid leukemia. Proc Natl Acad Sci USA. 2006;103(8):2794‐2799. 16477019 10.1073/pnas.0510423103 PMC1413797

[15] Willenbrock K , Kuppers R , Renne C , et al. Common features and differences in the transcriptome of large cell anaplastic lymphoma and classical Hodgkin's lymphoma. Haematologica. 2006;91(5):596‐604. 16670065

[16] Doolan P , Clynes M , Kennedy S , Mehta JP , Crown J , O'Driscoll L . Prevalence and prognostic and predictive relevance of PRAME in breast cancer. Breast Cancer Res Treat. 2008;109(2):359‐365. 17624586 10.1007/s10549-007-9643-3

[17] Oberthuer A , Hero B , Spitz R , Berthold F , Fischer M . The tumor‐associated antigen PRAME is universally expressed in high‐stage neuroblastoma and associated with poor outcome. Clin Cancer Res. 2004;10(13):4307‐4313. 15240516 10.1158/1078-0432.CCR-03-0813

[18] Steinbach D , Hermann J , Viehmann S , Zintl F , Gruhn B . Clinical implications of PRAME gene expression in childhood acute myeloid leukemia. Cancer Genet Cytogenet. 2002;133(2):118‐123. 11943337 10.1016/s0165-4608(01)00570-2

[19] Quintarelli C , Dotti G , De Angelis B , et al. Cytotoxic T lymphocytes directed to the preferentially expressed antigen of melanoma (PRAME) target chronic myeloid leukemia. Blood. 2008;112(5):1876‐1885. 18591381 10.1182/blood-2008-04-150045 PMC3401035

[20] Rezvani K , Yong AS , Tawab A , et al. Ex vivo characterization of polyclonal memory CD8+ T‐cell responses to PRAME‐specific peptides in patients with acute lymphoblastic leukemia and acute and chronic myeloid leukemia. Blood. 2009;113(10):2245‐2255. 18988867 10.1182/blood-2008-03-144071 PMC2652370

[21] Griffioen M , Kessler JH , Borghi M , et al. Detection and functional analysis of CD8+ T cells specific for PRAME: a target for T‐cell therapy. Clin Cancer Res. 2006;12(10):3130‐3136. 16707612 10.1158/1078-0432.CCR-05-2578

[22] Greiner J , Schmitt M , Li LI , et al. Expression of tumor‐associated antigens in acute myeloid leukemia: implications for specific immunotherapeutic approaches. Blood. 2006;108(13):4109‐4117. 16931630 10.1182/blood-2006-01-023127

[23] Greiner J , Ringhoffer M , Taniguchi M , et al. mRNA expression of leukemia‐associated antigens in patients with acute myeloid leukemia for the development of specific immunotherapies. Int J Cancer. 2004;108(5):704‐711. 14696097 10.1002/ijc.11623

[24] Bray F , Ferlay J , Soerjomataram I , Siegel RL , Torre LA , Jemal A . Global cancer statistics 2018: GLOBOCAN estimates of incidence and mortality worldwide for 36 cancers in 185 countries. CA Cancer J Clin. 2018;68(6):394‐424. 30207593 10.3322/caac.21492

[25] Tessari A , Pilla L , Silvia D , et al. Expression of NY‐ESO‐1, MAGE‐A3, PRAME and WT1 in different subgroups of breast cancer: an indication to immunotherapy? Breast. 2018;42:68‐73. 30189381 10.1016/j.breast.2018.08.106

[26] Lacher MD , Bauer G , Fury B , et al. SV‐BR‐1‐GM, a clinically effective GM‐CSF‐secreting breast cancer cell line, expresses an immune signature and directly activates CD4(+) T lymphocytes. Front Immunol. 2018;9:776. 29867922 10.3389/fimmu.2018.00776 PMC5962696

[27] Sun Y , Goodison S , Li J , Liu L , Farmerie W . Improved breast cancer prognosis through the combination of clinical and genetic markers. Bioinformatics. 2007;23(1):30‐37. 17130137 10.1093/bioinformatics/btl543 PMC3431620

[28] Epping MT , Hart AA , Glas AM , Krijgsman O , Bernards R . PRAME expression and clinical outcome of breast cancer. Br J Cancer. 2008;99(3):398‐403. 18648365 10.1038/sj.bjc.6604494 PMC2527791

[29] Al‐Khadairi G , Naik A , Thomas R , Al‐Sulaiti B , Rizly S , Decock J . PRAME promotes epithelial‐to‐mesenchymal transition in triple negative breast cancer. J Transl Med. 2019;17(1):9. 30602372 10.1186/s12967-018-1757-3 PMC6317205

[30] Sun Y , Urquidi V , Goodison S . Derivation of molecular signatures for breast cancer recurrence prediction using a two‐way validation approach. Breast Cancer Res Treat. 2010;119(3):593‐599. 19291396 10.1007/s10549-009-0365-6 PMC2844120

[31] Sun Z , Wu Z , Zhang F , et al. PRAME is critical for breast cancer growth and metastasis. Gene. 2016;594(1):160‐164. 27632898 10.1016/j.gene.2016.09.016

[32] Tajeddine N , Gala JL , Louis M , Van Schoor M , Tombal B , Gailly P . Tumor‐associated antigen preferentially expressed antigen of melanoma (PRAME) induces caspase‐independent cell death in vitro and reduces tumorigenicity in vivo. Cancer Res. 2005;65(16):7348‐7355. 16103086 10.1158/0008-5472.CAN-04-4011

[33] Jia Y , Chng WJ , Zhou J . Super‐enhancers: critical roles and therapeutic targets in hematologic malignancies. J Hematol Oncol. 2019;12(1):77. 31311566 10.1186/s13045-019-0757-y PMC6636097

[34] Yang L , Wang YZ , Zhu HH , et al. PRAME gene copy number variation is related to its expression in multiple myeloma. DNA Cell Biol. 2017;36(12):1099‐1107. 28953414 10.1089/dna.2017.3951

[35] Ercolak V , Paydas S , Bagir E , et al. PRAME expression and its clinical relevance in Hodgkin's lymphoma. Acta Haematol. 2015;134(4):199‐207. 26044287 10.1159/000381533

[36] Mitsuhashi K , Masuda A , Wang YH , Shiseki M , Motoji T . Prognostic significance of PRAME expression based on immunohistochemistry for diffuse large B‐cell lymphoma patients treated with R‐CHOP therapy. Int J Hematol. 2014;100(1):88‐95. 24820636 10.1007/s12185-014-1593-z

[37] Luetkens T , Schafhausen P , Uhlich F , et al. Expression, epigenetic regulation, and humoral immunogenicity of cancer‐testis antigens in chronic myeloid leukemia. Leuk Res. 2010;34(12):1647‐1655. 20409582 10.1016/j.leukres.2010.03.039

[38] McElwaine S , Mulligan C , Groet J , et al. Microarray transcript profiling distinguishes the transient from the acute type of megakaryoblastic leukaemia (M7) in Down's syndrome, revealing PRAME as a specific discriminating marker. Br J Haematol. 2004;125(6):729‐742. 15180862 10.1111/j.1365-2141.2004.04982.x

[39] Oka Y , Tsuboi A , Nakata J , et al. Wilms' tumor gene 1 (WT1) Peptide vaccine therapy for hematological malignancies: from CTL epitope identification to recent progress in clinical studies including a cure‐oriented strategy. Oncol Res Treat. 2017;40(11):682‐690. 29041012 10.1159/000481353

[40] Matsushita M , Ikeda H , Kizaki M , et al. Quantitative monitoring of the PRAME gene for the detection of minimal residual disease in leukaemia. Br J Haematol. 2001;112(4):916‐926. 11298586 10.1046/j.1365-2141.2001.02670.x

[41] Pellat‐Deceunynck C , Mellerin M‐P , Labarrière N , et al. The cancer germ‐line genes MAGE‐1, MAGE‐3 and PRAME are commonly expressed by human myeloma cells. Eur J Immunol. 2000;30(3):803‐809. 10741395 10.1002/1521-4141(200003)30:3<803::AID-IMMU803>3.0.CO;2-P

[42] Santamaría CM , Chillón MC , García‐Sanz R , et al. Molecular stratification model for prognosis in cytogenetically normal acute myeloid leukemia. Blood. 2009;114(1):148‐152. 19398719 10.1182/blood-2008-11-187724

[43] Tanaka N , Wang YH , Shiseki M , Takanashi M , Motoji T . Inhibition of PRAME expression causes cell cycle arrest and apoptosis in leukemic cells. Leuk Res. 2011;35(9):1219‐1225. 21550659 10.1016/j.leukres.2011.04.005

[44] De Carvalho DD , Binato R , Pereira WO , et al. BCR‐ABL‐mediated upregulation of PRAME is responsible for knocking down TRAIL in CML patients. Oncogene. 2011;30(2):223‐233. 20838376 10.1038/onc.2010.409

[45] Santamaria C , Chillon MC , Garcia‐Sanz R , et al. The relevance of preferentially expressed antigen of melanoma (PRAME) as a marker of disease activity and prognosis in acute promyelocytic leukemia. Haematologica. 2008;93(12):1797‐1805. 18815192 10.3324/haematol.13214

[46] Zhang Y‐H , Lu A‐D , Yang LU , et al. PRAME overexpression predicted good outcome in pediatric B‐cell acute lymphoblastic leukemia patients receiving chemotherapy. Leuk Res. 2017;52:43‐49. 27875783 10.1016/j.leukres.2016.11.005

[47] Xu Y , Rong LJ , Meng SL , Hou FL , Zhang JH , Pan G . PRAME promotes in vitro leukemia cells death by regulating S100A4/p53 signaling. Eur Rev Med Pharmacol Sci. 2016;20(6):1057‐1063. 27049257

[48] Xu Y , Yue Q , Wei H , Pan G . PRAME induces apoptosis and inhibits proliferation of leukemic cells in vitro and in vivo. Int J Clin Exp Pathol. 2015;8(11):14549‐14555. 26823776 PMC4713562

[49] Luznik L , Fuchs EJ . Donor lymphocyte infusions to treat hematologic malignancies in relapse after allogeneic blood or marrow transplantation. Cancer Control. 2002;9(2):123‐137. 11965233 10.1177/107327480200900205

[50] Dazzi F , Szydlo RM , Goldman JM . Donor lymphocyte infusions for relapse of chronic myeloid leukemia after allogeneic stem cell transplant: where we now stand. Exp Hematol. 1999;27(10):1477‐1486. 10517488 10.1016/s0301-472x(99)00096-x

[51] Giralt S , Hester J , Huh Y , et al. CD8‐depleted donor lymphocyte infusion as treatment for relapsed chronic myelogenous leukemia after allogeneic bone marrow transplantation. Blood. 1995;86(11):4337‐4343. 7492795

[52] Bleakley M , Otterud BE , Richardt JL , et al. Leukemia‐associated minor histocompatibility antigen discovery using T‐cell clones isolated by in vitro stimulation of naive CD8+ T cells. Blood. 2010;115(23):4923‐4933. 20203263 10.1182/blood-2009-12-260539 PMC2890170

[53] Biernacki MA , Marina O , Zhang W , et al. Efficacious immune therapy in chronic myelogenous leukemia (CML) recognizes antigens that are expressed on CML progenitor cells. Cancer Res. 2010;70(3):906‐915. 20103624 10.1158/0008-5472.CAN-09-2303 PMC2832197

[54] Quintarelli C , Dotti G , Hasan ST , et al. High‐avidity cytotoxic T lymphocytes specific for a new PRAME‐derived peptide can target leukemic and leukemic‐precursor cells. Blood. 2011;117(12):3353‐3362. 21278353 10.1182/blood-2010-08-300376 PMC3069675

[55] Kawahara M , Hori T , Matsubara Y , Okawa K , Uchiyama T . Identification of HLA class I‐restricted tumor‐associated antigens in adult T cell leukemia cells by mass spectrometric analysis. Exp Hematol. 2006;34(11):1496‐1504. 17046569 10.1016/j.exphem.2006.06.010

[56] Winkler C , Steingrube DS , Altermann W , et al. Hodgkin's lymphoma RNA‐transfected dendritic cells induce cancer/testis antigen‐specific immune responses. Cancer Immunol Immunother. 2012;61(10):1769‐1779. 22419371 10.1007/s00262-012-1239-z PMC11029013

[57] Staege MS , Banning‐Eichenseer U , Weißflog G , et al. Gene expression profiles of Hodgkin's lymphoma cell lines with different sensitivity to cytotoxic drugs. Exp Hematol. 2008;36(7):886‐896. 18400362 10.1016/j.exphem.2008.02.014

[58] Li LI , Reinhardt P , Schmitt A , et al. Dendritic cells generated from acute myeloid leukemia (AML) blasts maintain the expression of immunogenic leukemia associated antigens. Cancer Immunol Immunother. 2005;54(7):685‐693. 15627212 10.1007/s00262-004-0631-8 PMC11034334

[59] Liberante FG , Pellagatti A , Boncheva V , et al. High and low, but not intermediate, PRAME expression levels are poor prognostic markers in myelodysplastic syndrome at disease presentation. Br J Haematol. 2013;162(2):282‐285. 23594062 10.1111/bjh.12352

[60] Qin Y‐Z , Zhu H‐H , Liu Y‐R , et al. PRAME and WT1 transcripts constitute a good molecular marker combination for monitoring minimal residual disease in myelodysplastic syndromes. Leuk Lymphoma. 2013;54(7):1442‐1449. 23110703 10.3109/10428194.2012.743656

[61] Spanaki A , Perdikogianni C , Linardakis E , Kalmanti M . Quantitative assessment of PRAME expression in diagnosis of childhood acute leukemia. Leuk Res. 2007;31(5):639‐642. 16860864 10.1016/j.leukres.2006.06.006

[62] Mo XD , Qin YZ , Zhang XH , et al. Minimal residual disease monitoring and preemptive immunotherapy in myelodysplastic syndrome after allogeneic hematopoietic stem cell transplantation. Ann Hematol. 2016;95(8):1233‐1240. 27302479 10.1007/s00277-016-2706-y

[63] Atanackovic D , Luetkens T , Kloth B , et al. Cancer‐testis antigen expression and its epigenetic modulation in acute myeloid leukemia. Am J Hematol. 2011;86(11):918‐922. 21898529 10.1002/ajh.22141

[64] Bankovic J , Stojsic J , Jovanovic D , et al. Identification of genes associated with non‐small‐cell lung cancer promotion and progression. Lung Cancer. 2010;67(2):151‐159. 19473719 10.1016/j.lungcan.2009.04.010

[65] Pan SH , Su KY , Spiessens B , et al. Gene expression of MAGE‐A3 and PRAME tumor antigens and EGFR mutational status in Taiwanese non‐small cell lung cancer patients. Asia Pac J Clin Oncol. 2017;13(5):e212‐e223. 27519286 10.1111/ajco.12586

[66] Huang Q , Wei H , Wu Z , et al. Preferentially expressed antigen of melanoma prevents lung cancer metastasis. PLoS ONE. 2016;11(7):e0149640. 27391090 10.1371/journal.pone.0149640 PMC4938541

[67] Pujol JL , De Pas T , Rittmeyer A , et al. Safety and immunogenicity of the PRAME cancer immunotherapeutic in patients with resected non‐small cell lung cancer: a phase I dose escalation study. J Thorac Oncol. 2016;11(12):2208‐2217. 27544054 10.1016/j.jtho.2016.08.120

[68] Babiak A , Steinhauser M , Gotz M , Herbst C , Dohner H , Greiner J . Frequent T cell responses against immunogenic targets in lung cancer patients for targeted immunotherapy. Oncol Rep. 2014;31(1):384‐390. 24154794 10.3892/or.2013.2804

[69] De Pas T , Giovannini M , Rescigno M , et al. Vaccines in non‐small cell lung cancer: rationale, combination strategies and update on clinical trials. Crit Rev Oncol Hematol. 2012;83(3):432‐443. 22366114 10.1016/j.critrevonc.2011.12.005

[70] Cronin KA , Lake AJ , Scott S , et al. Annual report to the nation on the status of cancer, part I: national cancer statistics. Cancer. 2018;124(13):2785‐2800. 29786848 10.1002/cncr.31551 PMC6033186

[71] Beard RE , Abate‐Daga D , Rosati SF , et al. Gene expression profiling using nanostring digital RNA counting to identify potential target antigens for melanoma immunotherapy. Clin Cancer Res. 2013;19(18):4941‐4950. 24021875 10.1158/1078-0432.CCR-13-1253 PMC3778100

[72] Gezgin G , Luk SJ , Cao J , et al. PRAME as a potential target for immunotherapy in metastatic uveal melanoma. JAMA Ophthalmol. 2017;135(6):541‐549. 28448663 10.1001/jamaophthalmol.2017.0729 PMC5509351

[73] Schefler AC , Koca E , Bernicker EH , Correa ZM . Relationship between clinical features, GEP class, and PRAME expression in uveal melanoma. Graefes Arch Clin Exp Ophthalmol. 2019;257(7):1541‐1545. 31065847 10.1007/s00417-019-04335-w

[74] Field MG , Durante MA , Decatur CL , et al. Epigenetic reprogramming and aberrant expression of PRAME are associated with increased metastatic risk in Class 1 and Class 2 uveal melanomas. Oncotarget. 2016;7(37):59209‐59219. 27486988 10.18632/oncotarget.10962 PMC5312306

[75] Field MG , Decatur CL , Kurtenbach S , et al. PRAME as an independent biomarker for metastasis in uveal melanoma. Clin Cancer Res. 2016;22(5):1234‐1242. 26933176 10.1158/1078-0432.CCR-15-2071 PMC4780366

[76] Cai L , Paez‐Escamilla M , Walter SD , et al. Gene expression profiling and PRAME status versus tumor‐node‐metastasis staging for prognostication in uveal melanoma. Am J Ophthalmol. 2018;195:154‐160. 30092184 10.1016/j.ajo.2018.07.045 PMC6214741

[77] Sakurai E , Maesawa C , Shibazaki M , et al. Downregulation of microRNA‐211 is involved in expression of preferentially expressed antigen of melanoma in melanoma cells. Int J Oncol. 2011;39(3):665‐672. 21687938 10.3892/ijo.2011.1084

[78] Lee YK , Park UH , Kim EJ , Hwang JT , Jeong JC , Um SJ . Tumor antigen PRAME is up‐regulated by MZF1 in cooperation with DNA hypomethylation in melanoma cells. Cancer Lett. 2017;403:144‐151. 28634046 10.1016/j.canlet.2017.06.015

[79] Sigalotti L , Fratta E , Coral S , et al. Intratumor heterogeneity of cancer/testis antigens expression in human cutaneous melanoma is methylation‐regulated and functionally reverted by 5‐aza‐2'‐deoxycytidine. Cancer Res. 2004;64(24):9167‐9171. 15604288 10.1158/0008-5472.CAN-04-1442

[80] Gutzmer R , Rivoltini L , Levchenko E , et al. Safety and immunogenicity of the PRAME cancer immunotherapeutic in metastatic melanoma: results of a phase I dose escalation study. ESMO Open. 2016;1(4):e000068. 27843625 10.1136/esmoopen-2016-000068 PMC5070281

[81] Adib TR , Henderson S , Perrett C , et al. Predicting biomarkers for ovarian cancer using gene‐expression microarrays. Br J Cancer. 2004;90(3):686‐692. 14760385 10.1038/sj.bjc.6601603 PMC2409606

[82] Lancaster JM , Dressman HK , Whitaker RS , et al. Gene expression patterns that characterize advanced stage serous ovarian cancers. J Soc Gynecol Investig. 2004;11(1):51‐59. 10.1016/j.jsgi.2003.07.004 14706684

[83] Zhang WA , Barger CJ , Eng KH , et al. PRAME expression and promoter hypomethylation in epithelial ovarian cancer. Oncotarget. 2016;7(29):45352‐45369. 27322684 10.18632/oncotarget.9977 PMC5216727

[84] Kreuzinger C , von der Decken I , Wolf A , et al. Patient‐derived cell line models revealed therapeutic targets and molecular mechanisms underlying disease progression of high grade serous ovarian cancer. Cancer Lett. 2019;459:1‐12. 31150822 10.1016/j.canlet.2019.05.032

[85] Partheen K , Levan K , Österberg L , et al. Four potential biomarkers as prognostic factors in stage III serous ovarian adenocarcinomas. Int J Cancer. 2008;123(9):2130‐2137. 18709641 10.1002/ijc.23758

[86] Partheen K , Levan K , Osterberg L , Horvath G . Expression analysis of stage III serous ovarian adenocarcinoma distinguishes a sub‐group of survivors. Eur J Cancer. 2006;42(16):2846‐2854. 16996261 10.1016/j.ejca.2006.06.026

[87] Partheen K , Levan K , Osterberg L , Claesson I , Sundfeldt K , Horvath G . External validation suggests Integrin beta 3 as prognostic biomarker in serous ovarian adenocarcinomas. BMC Cancer. 2009;9:336. 19775429 10.1186/1471-2407-9-336 PMC2754489

[88] Wilky BA , Trucco MM , Subhawong TK , et al. Axitinib plus pembrolizumab in patients with advanced sarcomas including alveolar soft‐part sarcoma: a single‐centre, single‐arm, phase 2 trial. Lancet Oncol. 2019;20(6):837‐848. 31078463 10.1016/S1470-2045(19)30153-6

[89] Roszik J , Wang W‐L , Livingston JA , et al. Overexpressed PRAME is a potential immunotherapy target in sarcoma subtypes. Clin Sarcoma Res. 2017;7:11. 28630682 10.1186/s13569-017-0077-3 PMC5471883

[90] Oda Y , Yamamoto H , Kohashi K , et al. Soft tissue sarcomas: from a morphological to a molecular biological approach. Pathol Int. 2017;67(9):435‐446. 28759137 10.1111/pin.12565

[91] Iura K , Kohashi K , Hotokebuchi Y , et al. Cancer‐testis antigens PRAME and NY‐ESO‐1 correlate with tumour grade and poor prognosis in myxoid liposarcoma. J Pathol Clin Res. 2015;1(3):144‐159. 27499900 10.1002/cjp2.16 PMC4939879

[92] Hemminger JA , Toland AE , Scharschmidt TJ , Mayerson JL , Guttridge DC , Iwenofu OH . Expression of cancer‐testis antigens MAGEA1, MAGEA3, ACRBP, PRAME, SSX2, and CTAG2 in myxoid and round cell liposarcoma. Mod Pathol. 2014;27(9):1238‐1245. 24457462 10.1038/modpathol.2013.244 PMC4287229

[93] Tian W , Li Y , Zhang J , Li J , Gao J . Combined analysis of DNA methylation and gene expression profiles of osteosarcoma identified several prognosis signatures. Gene. 2018;650:7‐14. 29407229 10.1016/j.gene.2018.01.093

[94] Tan P , Zou C , Yong B , et al. Expression and prognostic relevance of PRAME in primary osteosarcoma. Biochem Biophys Res Commun. 2012;419(4):801‐808. 22390931 10.1016/j.bbrc.2012.02.110

[95] Altvater B , Kailayangiri S , Theimann N , et al. Common Ewing sarcoma‐associated antigens fail to induce natural T cell responses in both patients and healthy individuals. Cancer Immunol Immunother. 2014;63(10):1047‐1060. 24973179 10.1007/s00262-014-1574-3 PMC11028878

[96] Pollack SM , Li Y , Blaisdell MJ , et al. NYESO‐1/LAGE‐1s and PRAME are targets for antigen specific T cells in chondrosarcoma following treatment with 5‐Aza‐2‐deoxycitabine. PLoS ONE. 2012;7(2):e32165. 22384167 10.1371/journal.pone.0032165 PMC3288075

[97] Zhu H , Wang J , Yin J , et al. Downregulation of PRAME suppresses proliferation and promotes apoptosis in hepatocellular carcinoma through the activation of P53 mediated pathway. Cell Physiol Biochem. 2018;45(3):1121‐1135. 29439259 10.1159/000487353

[98] Szczepanski MJ , DeLeo AB , Luczak M , et al. PRAME expression in head and neck cancer correlates with markers of poor prognosis and might help in selecting candidates for retinoid chemoprevention in pre‐malignant lesions. Oral Oncol. 2013;49(2):144‐151. 22944049 10.1016/j.oraloncology.2012.08.005 PMC3607432

[99] Orlando D , Miele E , De Angelis B , et al. Adoptive immunotherapy using PRAME‐specific T cells in medulloblastoma. Cancer Res. 2018;78(12):3337‐3349. 29615432 10.1158/0008-5472.CAN-17-3140

[100] Xu B , Jungbluth AA , Frosina D , et al. The immune microenvironment and expression of PD‐L1, PD‐1, PRAME and MHC I in salivary duct carcinoma. Histopathology. 2019;75(5):672‐682. 31237963 10.1111/his.13944 PMC6812589

[101] Meng D , Yang S , Wan X , et al. A transcriptional target of androgen receptor, miR‐421 regulates proliferation and metabolism of prostate cancer cells. Int J Biochem Cell Biol. 2016;73:30‐40. 26827675 10.1016/j.biocel.2016.01.018

[102] Nettersheim D , Arndt I , Sharma R , et al. The cancer/testis‐antigen PRAME supports the pluripotency network and represses somatic and germ cell differentiation programs in seminomas. Br J Cancer. 2016;115(4):454‐464. 27441500 10.1038/bjc.2016.187 PMC4985348

[103] Dyrskjøt L , Zieger K , Kissow Lildal T , et al. Expression of MAGE‐A3, NY‐ESO‐1, LAGE‐1 and PRAME in urothelial carcinoma. Br J Cancer. 2012;107(1):116‐122. 22596240 10.1038/bjc.2012.215 PMC3389414

[104] Weber JS , Vogelzang NJ , Ernstoff MS , et al. A phase 1 study of a vaccine targeting preferentially expressed antigen in melanoma and prostate‐specific membrane antigen in patients with advanced solid tumors. J Immunother. 2011;34(7):556‐567. 21760528 10.1097/CJI.0b013e3182280db1 PMC3709852

